# Seroadaptive Practices: Association with HIV Acquisition among HIV-Negative Men Who Have Sex with Men

**DOI:** 10.1371/journal.pone.0045718

**Published:** 2012-10-03

**Authors:** Snigdha Vallabhaneni, Xin Li, Eric Vittinghoff, Deborah Donnell, Christopher D. Pilcher, Susan P. Buchbinder

**Affiliations:** 1 Department of Medicine, University of California, San Francisco, California, United States of America; 2 Fred Hutchinson Cancer Research Center, Seattle, Washington, United States of America; 3 Department of Epidemiology and Biostatistics, University of California, San Francisco, California, United States of America; 4 San Francisco Department of Public Health, San Francisco, California, United States of America; Public Health Agency of Barcelona, Spain

## Abstract

**Background:**

Although efficacy is unknown, many men who have sex with men (MSM) attempt to reduce HIV risk by adapting condom use, partner selection, or sexual position to the partner’s HIV serostatus. We assessed the association of seroadaptive practices with HIV acquisition.

**Methodology/Principal Findings:**

We pooled data on North American MSM from four longitudinal HIV-prevention studies. Sexual behaviors reported during each six-month interval were assigned sequentially to one of six mutually exclusive risk categories: (1) no unprotected anal intercourse (UAI), (2) having a single negative partner, (3) being an exclusive top (only insertive anal sex), (4) serosorting (multiple partners, all HIV negative), (5) seropositioning (only insertive anal sex with potentially discordant partners), and (6) UAI with no seroadaptive practices. HIV antibody testing was conducted at the end of each interval. We used Cox models to evaluate the independent association of each category with HIV acquisition, controlling for number of partners, age, race, drug use, and intervention assignment. 12,277 participants contributed to 60,162 six-month intervals with 663 HIV seroconversions. No UAI was reported in 47.4% of intervals, UAI with some seroadaptive practices in 31.8%, and UAI with no seroadaptive practices in 20.4%. All seroadaptive practices were associated with a lower risk, compared to UAI with no seroadaptive practices. However, compared to no UAI, serosorting carried twice the risk (HR = 2.03, 95%CI:1.51–2.73), whereas seropositioning was similar in risk (HR = 0.85, 95%CI:0.50–1.44), and UAI with a single negative partner and as an exclusive top were both associated with a lower risk (HR = 0.56, 95%CI:0.32–0.96 and HR = 0.55, 95%CI:0.36–0.84, respectively).

**Conclusions/Significance:**

Seroadaptive practices appear protective when compared with UAI with no seroadaptive practices, but serosorting appears to be twice as risky as no UAI. Condom use and limiting number of partners should be advocated as first-line prevention strategies, but seroadaptive practices may be considered harm-reduction for men at greatest risk.

## Introduction

Seroadaptation means modifying sexual practices based on the perceived HIV serostatus of a sexual partner [1], motivated by the obvious fact that HIV transmission can only occur in a serodiscordant pairing, and abundant evidence that transmission risk in serodiscordant unprotected anal sex (UAI) is lower if the HIV-negative partner is insertive [2]. For HIV-negative men who have sex with men (MSM), serosorting is engaging in UAI only with partners perceived to be HIV-negative, and seropositioning is taking the insertive role in serodiscordant UAI.

Seroadaptive practices originated within communities at risk for HIV, and have been increasingly reported in many countries [3]–[6]. Among MSM, seroadaptive practices may be more common and more consistently adhered to than condom use, and appear to be deliberately adopted with the intention to reduce HIV risk [7], [8]. However, these practices remain controversial due to unproven efficacy. Prior research suggests that while serosorting may achieve reductions in risk relative to no seroadaptive practices at all (i.e, no partner selection, no sexual position preference, and no condom use), it is nonetheless riskier than not having any UAI [5], [9]–[11]. The likely explanation is that serosorting is vulnerable to misperception of partner serostatus. In a longitudinal study of 4295 MSM in the US conducted in the late 1990′s, one-fifth of new HIV infections could be attributed to receptive UAI with a partner thought to be HIV negative [12]. Furthermore, modeling studies suggest that any potential benefits of serosorting could be undermined by undiagnosed HIV infection, particularly among partners in the acute stage when the HIV antibody is undetectable and infectiousness is relatively high [13], [14]. Seropositioning may also not be very effective because serodiscordant UAI still poses some risk to an insertive HIV-negative partner [15].

To assess the efficacy of these behaviors, we evaluated the independent association between seroadaptive practices and HIV acquisition in a large prospective cohort of HIV-negative North American MSM.

## Methods

### Sources of Data

We pooled data from four longitudinal HIV prevention studies of HIV-uninfected MSM conducted from 1995–2007. The HIVNET Vaccine Preparedness Study (VPS) (1995–1998), was an observational study of HIV risk behaviors and seroincidence [16]. VAX004 (1998–2001; ClinicalTrials.gov/NCT00002441), was a randomized controlled trial (RCT) of an HIV vaccine, which showed no efficacy at preventing HIV infection [17]. EXPLORE (1999–2003; ClinicalTrials.gov/NCT00000931), was an RCT of a behavioral intervention, which showed modest reductions in self-reported risk behavior, but no statistically significant reduction in HIV acquisition [18]. Finally, STEP (2004–2007; ClinicalTrials.gov/NCT00095576), an RCT of another HIV vaccine, was stopped early when an interim analysis met pre-specified futility boundaries [19]. Although there was some variability in specific enrollment criteria, all of the studies sought to enroll men who reported, at the very least, anal sex with one or more men in the past 12 months, VPS, VAX004 and STEP also enrolled participants from other continents or risk groups, however in this analysis, we include only North American MSM.

All four studies followed participants every six months for 18–48 months. Sexual behavior over the last six months was assessed at each visit. VPS, VAX004, and STEP used face-to-face interviews, while EXPLORE used audio-computer-assisted self interview (ACASI). Sexual history obtained at each visit included total number of sex partners, perceived serostatus of each partner, and occurrence of specific sexual practices (insertive/receptive anal sex and protected/unprotected anal sex) with partners of each serostatus. Information on any methamphetamine or amyl nitrite (popper) use was also obtained. Questions regarding sexual behavior were asked in a similar format in these studies, enabling us to categorize each participant visit into one of the six seroadaptive behavior categories described below. Finally, HIV-antibody testing was conducted at the time of each interview.

### Categorization of Seroadaptive Practices

Over the duration of the study, reported behaviors in the previous six months were categorized sequentially within each study interval into the following six mutually exclusive, categories:

#### 1. No UAI

intervals during which participants reported no sex of any kind, only oral sex, and consistent condom use during all anal sex episodes, regardless of partner serostatus.

#### 2. Single negative partner

intervals during which participants reported UAI but only with one anal sex partner, believed to be HIV negative.

#### 3. Exclusive top

intervals during which participants reported some UAI but always as the insertive partner, regardless of serostatus of the partner.

#### 4. Pure serosorting

intervals during which participants reported both insertive and receptive UAI with multiple partners, but all were believed to be HIV negative.

#### 5. Pure seropositioning

intervals during which participants reported some UAI and had some HIV-positive or unknown-serostatus partners, but reported only insertive anal intercourse with these potentially discordant partners. Participants could have reported insertive or receptive anal intercourse with partners they believed to be HIV negative.

#### 6. No seroadaptive behavior

all other intervals, during which participants reported some UAI without potentially protective seroadaptive practices and engaged in receptive anal intercourse with an HIV-positive or unknown-status partner.

Only the first category includes no UAI, while categories 2–5 represent some form of seroadaptation. Unprotected receptive oral sex with ejaculation was allowed in the no UAI category because it is lower risk than any form of anal sex, with the possible exception of protected insertive anal sex [2]. Over the duration of the study, participants could be placed into a different category at each study interval, based on their reported behaviors in the previous six months.

In order to evaluate a wider range of seroadaptive practices that have been reported in the literature, we then undertook a secondary analysis, where we considered only unprotected sex acts in defining categories 3–6. Specifically,

#### 3. Unprotected top

intervals during which participants reported only unprotected insertive anal sex episodes; receptive anal sex with partner of any serostatus, if reported, was protected. Unlike the exclusive top group in the first categorization, where participants did not have any receptive anal sex, in this categorization, participants could report receptive anal sex, but condoms were used with all receptive sex episodes.

#### 4. Condom serosorting

intervals during which participants reported all unprotected UAI occurred exclusively with HIV-negative partners; any anal intercourse with HIV-positive or unknown-status partners was protected. This differs from pure serosorting, which did not allow any serodiscordant partners.

#### 5. Condom seropositioning

intervals during which participants reported unprotected receptive anal sex exclusively with HIV-negative partners; any receptive anal sex with HIV-positive or unknown–status partners was protected. In the pure seropositioning category, participants did not report any receptive anal sex with potentially discordant partners.

#### 6. Highest risk sex

intervals during which participants reported unprotected receptive anal intercourse with HIV-positive or unknown-status partners. In the no seroadaptive behavior category, participants reported UAI of some kind with potentially serodiscordant partners but receptive anal intercourse with such partners could have been protected or unprotected.

Finally, we conducted an additional sensitivity analysis in which these categorizations were defined using behaviors reported over 12 rather than 6 months, during the preceding as well as the current interval.

### Data Analysis

To assess the association between the various sexual risk categories and HIV infection, we used Cox models with the baseline hazard stratified by study. HIV acquisition was assessed at each six-month visit. Seroadaptive category was treated as a time-dependent covariate (TDC), with results summarized by relative hazards using category 1 and then category 6 as the reference level. Motivated by a priori substantive considerations, all models adjusted for intervention assignment in the studies that included an intervention arm, age at enrollment, calendar time at enrollment, and race/ethnicity as fixed covariates, as well as number of sexual partners and any methamphetamine or popper use in the prior six months as TDCs. We assessed modification of the effects of seroadapation category by these six covariates. Data analysis used SAS Version 9.2 (SAS Institute, Cary NC) and Stata Version 12.2 (Stata Corp, College Station, TX).

## Results

A total of 12,277 HIV-negative MSM from North America were included in the analysis. We observed 663 HIV seroconversions. Demographic and behavioral characteristics of participants at their baseline visit are presented in Table 1. The median age was 34, and more than three-quarters were White. At their baseline visit, 42.6% of participants reported six or more sexual partners in the prior six months. Ten percent reported methamphetamine use, and 29.0% reported “popper” use in the past six months. Participants were similar across cohorts, with two exceptions: first, VAX004 participants were more likely to be White (85.9%) and STEP participants reported lower levels of methamphetamine (5.9%) and popper (16.4%) use at baseline.

**Table 1:** Demographic and behavioral characteristics at baseline of North American men who have sex with men from four longitudinal cohort studies, 1995–2007.

Characteristic at baseline	VPS N (%)	Vax004 N(%)	Explore N(%)	STEP N(%)	Total N (%)
Total number of participants included	2974	4456	3798	1049	12,277
Median age (IQR)	32 (27–38)	36 (30–43)	33 (28–39)	34 (27–40)	34 (28–40)
Race					
White	2259 (76.0%)	3827 (85.9%)	2800 (73.7%)	754 (71.9%)	9640 (78.5%)
Black	183 (6.2%)	162 (3.6%)	241 (6.4%)	94 (9.0%)	680 (5.5%)
Hispanic	370 (12.4%)	295 (6.6%)	543 (14.3%)	133 (12.7%)	1341 (10.9%)
Asian	92 (3.1%)	71 (1.6%)	99 (2.6%)	32 (3.1%)	294 (2.4%)
Other	70 (2.4%)	101 (2.3%)	115 (3.0%)	36 (3.4%)	322 (2.6%)
Number of Partner in last 6 months					
0	0 (0.0%)	128 (2.8%)	83 (2.2%)	55 (5.2%)	266 (2.2%)
1	674 (22.7%)	986 (22.1%)	454 (12.0%)	226 (21.5%)	2340 (19.1%)
2–5	1117 (37.6%)	1669 (37.4%)	1273 (33.5%)	387 (36.9%)	4446 (36.2%)
6–10	535 (18.0%)	761 (17.1%)	791 (20.8%)	167 (15.9%)	2254 (18.4%)
>10	648 (21.8%)	912 (20.5%)	1197 (31.5%)	214 (20.4%)	2971 (24.2%)
Any methamphetamine use in thelast six months	272 (9.15%)	371 (8.3%)	479 (12.6%)	62 (5.9%)	1184 (9.6%)
Any popper use in the last six months	821 (27.6%)	1276 (28.6%)	1292 (34.0%)	172 (16.4%)	3561 (29.0%)

The 12,277 participants contributed 60,162 six-month intervals; 2,048 (4.0%) intervals were excluded from the analysis due to missing data on one or more of the covariates used in our models. Nearly half (47.4%) of all intervals were categorized as no UAI based on participant report of no anal sex of any kind, only oral sex, or 100% condom use with all anal sex episodes during the preceding six months (Table 2). Thirty-two percent of visits were categorized as UAI with some seroadaptive practices and the remaining 20.4% percent of intervals were categorized as UAI with no seroadaptive behavior. Although only one-third of intervals were characterized by seroadaptive practices, 62% of participants reported seroadaptive practices of some kind in at least one interval. Twenty-three percent (1784/7756) of participants with five or more intervals reported behavior that consistently fell into the same category across all visits. Seroconversions were observed in each category, and ranged from 0.25–2.95% per visit.

**Table 2:** Adjusted relative hazards of HIV seroconversion (without accounting for condoms) among North American men who have sex with men from four longitudinal cohort studies, 1995–2007.

				Model with No UAI as reference		Model with No seroadaptive behavior as reference##	
Risk category	Visits N (%)	HIV SC* (N)	SC per-cent	Adjusted HR**	95% CI	Adjusted HR	95% CI
No UAI	28,316 (47.6)	173	0.61	**reference**	**n/a**	0.31	0.25–0.37
Single negative partner	6393 (10.8)	16	0.25	0.56	0.32–0.96	0.17	0.10–0.30
Exclusive top	6169 (10.4)	25	0.40	0.55	0.36–0.84	0.17	0.11–0.25
Pure serosorting	4437 (7.5)	65	1.44	2.03	1.51–2.73	0.62	0.47–0.82
Pure Seropositioning	2048 (3.4)	15	0.73	0.85	0.50–1.44	0.26	0.15–0.43
No seroadaptivebehavior	12,136 (20.4)	369	2.95	3.27	2.68–3.99	**reference**	**n/a**

* seroconversion.

** adjusted for age, race, number of sexual partners, any methamphetamine and popper use in the last six months, and intervention assignment.

## models 1 and 2 are equivalent apart from using different groups as the reference category.

Using no seroadaptive behavior as the reference category, we observed substantial, statistically significant adjusted reductions in risk of HIV acquisition in intervals in which any of the four seroadaptive practices were reported (Table 2). Specifically, we observed a 38% reduction (HR: 0.62, 95% CI: 0.47–0.82) with pure serosorting, a 74% reduction (HR: 0.26, 95% CI: 0.15–0.43) with pure seropositioning, and 83% reductions with both single negative partner (HR: 0.17, 95% CI: 0.10–0.30) and exclusive top (HR: 0.17, 95% CI: 0.11–0.25) categories. Adjusted risk was also 69% lower (HR: 0.31, 95% CI: 0.25–0.37) in intervals where no UAI was reported. Equivalently, using no UAI as the reference category, pure serosorting was associated with a doubling of risk (HR: 2.03, 95% CI: 1.51–2.73). In addition, UAI with a single negative partner (HR: 0.56, 95% CI: 0.32–0.96) and UAI as an exclusive top (HR: 0.55, 95% CI: 0.36–0.84) were both associated with significantly lower risk.

In the secondary analysis in which only unprotected sex acts were used in defining categories 3–6, all four seroadaptive practices were again associated with substantially and statistically significant reductions risk, compared with highest risk sex. When using no UAI as the reference category, both condom serosorting (HR: 1.82, 95% CI: 1.39–2.37) and condom seropositioning (HR: 1.86, 95% CI: 1.21–2.86) were associated with substantial increases in risk (Table 3). Risk was similar in intervals categorized as unprotected top and no UAI (HR: 0.79, 95% CI: 0.58–1.07).

**Table 3:** Adjusted relative hazards of HIV seroconversion (with condoms) among North American men who have sex with men from four longitudinal cohort studies, 1995–2007.

				Model with No UAI as reference		Model with Highest risk sex as reference	
Risk category	Visits N (%)	HIV SC* (N)	SC per-cent	Adjusted HR**	95% CI	Adjusted HR	95% CI
No UAI	28316 (47.6)	173	0.61	**reference**	**n/a**	0.24	0.19–0.29
Single negative partner	6393 (10.8)	16	0.25	0.57	0.33–0.98	0.14	0.08–0.24
Unprotected top	9459 (15.9)	58	0.61	0.79	0.58–1.07	0.19	0.14–0.25
Condom serosorting	6287 (10.6)	88	1.38	1.82	1.39–2.37	0.43	0.34–0.55
Condom seropositioning	1357 (2.3)	25	1.81	1.86	1.21–2.86	0.44	0.29–0.67
Highest risk sex	7680 (12.9)	303	3.80	4.20	3.42–5.15	**reference**	**n/a**

* seroconversion.

** adjusted for age, race, number of sexual partners, and any methamphetamine and popper use in the last six months, and intervention assignment.

## models are equivalent except for using different groups as the reference category.

In additional sensitivity analyses, results were unchanged in analyses categorizing behaviors over the last 12 rather than last 6 months. We found no persuasive evidence of modification of the effects of seroadaptive behavior category by covariates, with one exception: the protective effect of no UAI, relative to no seroadaptive behavior, increased with age (HR: 0.86 per 5 years, 95% CI: 0.77–0.96), p = 0.006). Finally, there was no evidence of interaction between study and seroadaptive categories (p = 0.77).

## Discussion

In this analysis of over 12,000 North American MSM with more than 60,000 six-month intervals of follow-up and 663 seroconversions, we found that both pure and condom serosorting (having UAI with multiple HIV-negative partners, but no UAI with HIV-positive partners), and condom seropositioning (reporting condom use in all receptive anal sex with an HIV-positive or unknown-serostatus partner, and unprotected insertive anal sex with these partners) placed men at increased risk for HIV acquisition, compared with those reporting no UAI with partners of any serostatus. However, these and all other seroadaptive practices were significantly less risky than reporting receptive anal sex with HIV-positive or unknown-serostatus partners, with or without a condom. Men reporting only one HIV-negative partner or always being the insertive partner, regardless of condom use, were at the lowest risk.

It should be noted that all sexual practices in these cohorts of MSM carried some risk, as men in each of the categories, whether or not condom use was taken into account, had seroconversions. In fact, men reported no unprotected anal sex in the majority of intervals, and 173 of the 663 seroconversions occurred during the intervals when men reported no unprotected anal sex. This is likely a result of under-reporting of unprotected anal sex, in part due to social desirability bias, under-reporting of intermitted condom use and/or failure, and potentially some episodes of HIV acquisition from oral sex.

Definitions of various seroadaptive practices continue to evolve in the literature. Our pure [4] and condom [5] serosorting definitions have both been used previously. We did not find meaningful differences between these two categorizations. Our estimates of the risks associated with both definitions are similar to a recent report by Kennedy et. al. [20] of cohorts of MSM from the US and Australia [5], [9], [10]. In that meta-analysis, the summary odds-ratio for serosorting was 1.80 (95% CI: 1.21–2.70) relative to consistent condom use, and 0.46 (95% CI: 0.25–0.83) relative to no condom use and no partner selection.

In our study, having only insertive sex (even with a potentially serodiscordant partner) appeared to be safer than no UAI. Other studies have found similar risk for this practice, sometimes also called strategic positioning [21], as for no UAI [10]. Reasons for these findings are likely two-fold. First, per-contact risk for insertive anal sex is an order of magnitude lower than for receptive anal sex [2]. Second, some intervals were almost surely misclassified as no UAI, because of the under-reporting of UAI and unrecognized condom failure.

Having UAI with a single negative partner was associated with an even lower risk of HIV seroconversion than reporting no UAI. This finding underscores the importance of minimizing number of partners as a primary HIV prevention strategy. The cohorts we studied did not collect the data needed to assess another related seroadaptive practice called negotiated safety, defined as UAI with a steady seroconcordant partner, but only protected sex outside of this relationship. An Australian study found reduced risk among MSM reporting strict adherence to negotiated safety when compared to having no seroadaptive strategy at all [10].

The effectiveness of seroadaptive practices is dependent on accurate knowledge of serostatus and proper disclosure of serostatus, both of which are in turn dependent on frequent HIV testing. In the United States, an estimated 20% of those infected with HIV do not know their status [22]. Expanded testing initiatives, including home testing, may be key to improving testing frequency, and thus improving the effectiveness of seroadaptive practices.

Black MSM are disproportionately affected by HIV. Although they are equally likely to report seroadaptive behavior as White MSM, there is concern that seroadaptive behavior may not be as effective in this group [23] because of the high rates of undiagnosed HIV infection, lower reported serodisclosure, and assortative mixing in a community with high background HIV prevalence [24]. In our study, we did not find any evidence of effect modification by race. Encouragingly, Marks *et. al*, also found that serosorting was an effective risk reduction strategy among Black MSM, when compared to UAI with no seroadaptive strategies [9]. Further research on the prevalence and effectiveness of seroadaptive practices in this vulnerable population is needed.

We did not examine the impact of seroadaptive practices on sexually transmitted infections (STIs) in our cohort. However, other groups have show that there is an increased risk of bacterial STI among men who report engaging in seroadaptive practices compared to those who report no UAI at all [25]. STI risk should also be taken into consideration when weighing the advantages and disadvantages of seroadaptive practices.

There are several limitations to this study. The cohorts included in this analysis were followed over a decade, between 1995 and 2004, when HIV testing and treatment were changing rapidly. However, calendar time did not modify our findings for seroadaptive categories. In addition, our results were obtained using self-reported behavior, which despite the use of ACASI in one of the included cohorts, is subject to desirability bias as well as random errors; this probably explains the sizable number of seroconversions in intervals where no UAI was reported. Finally, because there was a 6–12 week window between HIV infection and seroconversion with the earlier generations of HIV antibody testing used in these studies, some seroconversions may have been misattributed to a later six-month interval, with potentially a different risk category. However, results were unchanged in analyses categorizing behaviors over the last 12 rather than last 6 months.

Another limitation is that we did not have the data to determine whether participants *intentionally* adopted one or more seroadaptive practices. Among the 25% of men classified into the same seroadaptive category across all of five or more follow up visits, we may have some indirect evidence of intention, but this pattern was uncommon. In general, individuals made multiple transitions between the six behavioral categories – variability which prevention interventions and counseling messages need to take into account. MSM intentionally adopting seroadaptive practices may be different than those reporting apparently seroadaptive behavior adopted for other reasons, making our results harder to interpret. The links between seroadaptive intention, uptake, maintenance, and risk are a much-needed area of study.

Our study also had several strengths. The four included cohorts were all large, at moderate to high risk, and followed for 18–48 months, providing good statistical power. In addition the sample was recruited in several US cities, enhancing representativeness. Finally, the questionnaires used were similar, enabling us to categorize behavior uniformly.

Given the increased risk we observed for some seroadaptive practices, compared to no UAI, we conclude that condom use and limiting number of partners should continue to be advocated as first-line HIV prevention strategies. Seroadaptive practices may be considered as harm-reduction strategies for MSM engaging in extremely high-risk behavior, but should not be recommended as primary prevention strategies. Finally, behavioral interventions need to be ongoing and tailored since individual risk behavior appears to vary substantially over time.

## Supporting Information

Table S1 **Adjusted relative hazards of HIV seroconversion among North American men who have sex with men from four longitudinal cohort studies, 1995–2007.**
(DOCX)
