# Supplementary material for: Seroadaptive Practices: Association with HIV Acquisition among HIV-Negative Men Who Have Sex with Men
Source: PLoS One. 2012 Oct 3;7(10):e45718. doi: 10.1371/journal.pone.0045718 (PMC3463589; doi:10.1371/journal.pone.0045718)
Supplement: Table S1 — Adjusted relative hazards of HIV seroconversion among North American men who have sex with men from four longitudinal cohort studies, 1995–2007. (DOCX) [file pone.0045718.s001.docx]

**Appendix 1**: **Adjusted relative hazards of HIV seroconversion among North American men who have sex with men from four longitudinal cohort studies, 1995-2007**

| Covariate | Adjusted hazard ratio* | 95% Confidence Interval |
| --- | --- | --- |
| Age | 0.98 | 0.97 - 0.99 |
| Race |  |  |
| White | reference |  |
| Black | 1.76 | 1.28 - 2.41 |
| Hispanic | 1.27 | 1.00 - 1.61 |
| Asian | 0.82 | 0.47 - 1.42 |
| Other | 1.57 | 1.06 - 2.32 |
| Number of sexual partners in the last 6 months |  |  |
| 0 | reference |  |
| 1 | 0.70 | 0.39 - 1.28 |
| 2-5 | 0.87 | 0.50 - 1.54 |
| 6-10 | 1.07 | 0.60 - 1.91 |
| >10 | 1.46 | 0.83 - 2.58 |
| Methamphetamine use in the last 6 months | 2.03 | 1.69-2.44 |
| “Popper” use in the last  6 months | 1.41 | 1.19-1.66 |
| Treatment assignment |  |  |
| No intervention/treatment | reference |  |
| Explore | 0.83 | 0.63 - 1.09 |
| Vax 004 | 0.94 | 0.74 - 1.18 |
| STEP | 1.39 | 0.84 - 2.27 |

*also adjusted for seroadaptive practices category; HRs for seroadaptive categories are presented in Table 2 and 3 of the manuscript.
